# The first isolation and molecular characterization of *Toxoplasma gondii* from horses in Serbia

**DOI:** 10.1186/s13071-017-2104-x

**Published:** 2017-04-04

**Authors:** Ivana Klun, Aleksandra Uzelac, Isabelle Villena, Aurélien Mercier, Branko Bobić, Aleksandra Nikolić, Irena Rajnpreht, Marieke Opsteegh, Dominique Aubert, Radu Blaga, Joke van der Giessen, Olgica Djurković-Djaković

**Affiliations:** 1 National Reference Laboratory for Toxoplasmosis, Centre of Excellence for Food- and Vector-Borne Zoonoses, Institute for Medical Research, University of Belgrade, Dr Subotića 4, 11129 Belgrade, Serbia; 2 Centre National de Référence de la Toxoplasmose, Laboratoire de Parasitologie-Mycologie, CHU Maison Blanche, EA 3800 SFR CAP-SANTE, UFR Médecine Université de Reims Champagne-Ardenne, 45 rue Cognacq-Jay, 51092 Reims, France; 3 INSERM, UMR_S 1094, Neuroépidémiologie Tropicale, Université de Limoges, 2 rue du Docteur Marcland, 87025 Limoges, France; 4 Toxoplasma Biological Resource Center (BRC), Centre Hospitalier-Universitaire Dupuytren, 87042 Limoges, France; 5 National Institute for Public Health and the Environment (RIVM), Antonie van Leeuwenhoeklaan 9, 3520BA Bilthoven, Netherlands; 6 Ecole Nationale Vétérinaire d’Alfort, UMR BIPAR, ANSES, INRA, Université Paris-Est, Laboratoire de santé animale de Maisons-Alfort, Maisons-Alfort, France

**Keywords:** *Toxoplasma gondii*, Horse, Serbia, Prevalence, Strain, Isolation, Genotyping, Microsatellite markers, Type III

## Abstract

**Background:**

Consumption of undercooked or insufficiently cured meat is a major risk factor for human infection with *Toxoplasma gondii*. Although horsemeat is typically consumed rare or undercooked, information on the risk of *T. gondii* from infected horse meat to humans is scarce. Here, we present the results of a study to determine the presence of *T. gondii* infection in slaughter horses in Serbia, and to attempt to isolate viable parasites.

**Methods:**

The study included horses from all regions of Serbia slaughtered at two abattoirs between June 2013 and June 2015. Blood sera were tested for the presence of specific IgG *T. gondii* antibodies by the modified agglutination test (MAT), and samples of trypsin-digested heart tissue were bioassayed in mice. Cyst-positive mouse brain homogenates were subjected to DNA extraction and *T. gondii* strains were genotyped using 15 microsatellite markers (MS).

**Results:**

A total of 105 slaughter horses were sampled. At the 1:6 cut-off 48.6% of the examined horses were seropositive, with the highest titre being 1:400. Viable parasites were isolated from two grade type mares; both parasite isolates (RS-Eq39 and RS-Eq40) were *T. gondii* type III, and both displayed an increased lethality for mice with successive passages. These are the first cases of isolation of *T. gondii* from horses in Serbia. When compared with a worldwide collection of 61 type III and type III-like strains, isolate RS-Eq39 showed a combination of MS lengths similar to a strain isolated from a duck in Iran, and isolate RS-Eq40 was identical in all markers to three strains isolated from a goat from Gabon, a sheep from France and a pig from Portugal. Interestingly, the source horses were one seronegative and one weakly seropositive.

**Conclusions:**

The isolation of viable *T. gondii* parasites from slaughter horses points to horsemeat as a potential source of human infection, but the fact that viable parasites were isolated from horses with only a serological trace of *T. gondii* infection presents further evidence that serology may not be adequate to assess the risk of toxoplasmosis from horsemeat consumption. Presence of *T. gondii* type III in Serbia sheds more light into the potential origin of this archetypal lineage in Europe.

**Electronic supplementary material:**

The online version of this article (doi:10.1186/s13071-017-2104-x) contains supplementary material, which is available to authorized users.

## Background

*Toxoplasma gondii* is considered one of the most successful parasites on Earth due to its omnipresence and widest array of hosts, including all mammals [1]. The genus comprises a single species infective for all hosts, with limited genetic diversity in Europe and North America where the majority of isolates belong to the clonal genotypes type II and III, with the predominance of type II [2, 3]. However, a wider genetic diversity characterized by non-clonal, atypical strains is found in South America and Africa, and is thought to be related to the presence of diverse Felidae as the only definitive host in which sexual reproduction, and consequentially, genetic recombinations, occur [4, 5].

Human infection is widespread; it has been estimated that one third of the global population is infected [6]. However, infection is generally mild and self-limiting, except in population categories with an incompetent immune system, including the foetus and immunosuppressed individuals, in which it may cause life-threatening disease. Treatment options have not advanced much for decades and there is still no drug able to eliminate encysted parasites.

Consumption of undercooked or insufficiently cured meat is a major risk factor for human infection with *T. gondii* [7–11].

Horsemeat is typically consumed rare or undercooked, particularly in some European countries such as Italy and France [12]. Serbia used to be an important horse producer and exporter, but the production has been significantly decreasing over the last decades. Local consumption of horsemeat currently constitutes about 0.01 kg *per capita* per year [13]. The presence of *T. gondii* in main meat producing animals in Serbia has been well established [14–16], but for horses there are very few data [14], and moreover, horse meat or tissues have not yet been analysed. Several strains originating from patients, as well as from pigs, sheep, rodents and pigeons have been already isolated and genotyped [17–20]. Viable cysts have been shown to persist for many months in various organs in experimentally infected horses [21].

Here, we present the results of a study to examine the presence of *T. gondii* infection in slaughter horses in Serbia, and to attempt to isolate viable parasites.

## Methods

### Study population and collection of samples

The total number of horses in Serbia is around 15,000 [22], of which about 400 are slaughtered each year. While no abattoir exclusively slaughters horses, those that do, usually slaughter them at a rate of once to twice per week. In this study, samples were obtained from horses slaughtered in two abattoirs, one in Northern Serbia and the other in the vicinity of Belgrade, between June 2013 and June 2015. The sampled materials included blood and heart tissue. The heart was chosen for sampling as it was identified as a predilection site [23]. Blood (8 ml) was collected in sterile centrifuge tubes (with no additive) during exsanguination at the slaughter line. The apex portion of the heart (at least 300 g) was collected into a sample bag. Each animal was designated with a unique ID number and the samples were labelled accordingly. Samples were transported on ice to the Serbian National Reference Laboratory for Toxoplasmosis (NRL Toxo) immediately after collection. Upon arrival, each blood sample was centrifuged at 800× *g* for 20 min, and the collected sera were immediately frozen and kept at -80 °C until testing for *T. gondii* antibodies. Heart tissue was processed immediately or kept at +4 °C until processing the following day.

### Collection of zoographical data

Data on horses were collected from the accompanying health certificates and included age, gender and region of origin of the animal. Horses were classified by age as yearlings (1–2 years), young (3–4 years) or adult (5–9 years old). The regions of origin included Northern, Western, Central and Eastern Serbia, and “not determined”, for horses brought for slaughter by traders who kept them just for a short time after purchase. Determination of horse type or breed was based on direct observation of the horses prior to slaughter, and on interview of the horse trader when present; horses were classified as grade (no dominant breed trait; for general use or light work only), working (used for medium work, dominantly Bosnian horse or Nonius breed mixes), and working heavy-type cold blooded horses.

### Study design

Horse sera were sent to the French National Reference Centre for Toxoplasmosis (NRC Toxo) laboratory in Reims, where they were tested for specific IgG *T. gondii* antibodies. Heart samples were processed (at Serbian NRL Toxo) by trypsin digestion, and the digests inoculated into mice for bioassay. Following a 6-week observation period, the mice were euthanized, and sera tested for *T. gondii* antibodies, while brains were examined microscopically for *T. gondii* cysts. If cysts were observed, a portion of the brain homogenate was saved for DNA extraction for genotyping, and the rest re-inoculated into fresh mice for strain propagation. Finally, the DNA samples extracted from the mouse brain homogenates were sent to the Biological Resource Centre *Toxoplasma* (BRC) in Limoges for strain genotyping.

### Serology

*T. gondii*-specific IgG antibodies were detected by a modified agglutination test (MAT) as described by Desmonts and Remington [24], the antigen for which was prepared by NRC Toxo in Reims. Horse sera were tested at NRC Toxo, at starting dilutions of 1:6, 1:10 and 1:25, and two-fold dilutions thereafter if needed.

Mouse sera were tested at the NRL Toxo in Belgrade, using the same protocol, but starting at a 1:20 dilution.

### Trypsin digestion

Heart tissue samples were trimmed to remove any excess fat and connective tissue, and cut into roughly 2 × 2 cm cubes. A total of 200 g of tissue was weighed out and coarsely blended in a food processor. The minced tissue was digested with 0.25% porcine trypsin (T4674, Sigma-Aldrich, St. Louis, MO, USA) in sterile saline (final volume of 300 ml), supplemented with 200× penicillin-streptomycin solution (PAA Laboratories GmbH, Pasching, Austria), and 27 μg/ml amoxicillin (Hemofarm, Vršac, Serbia). The digestion was performed at 37 °C for 1.5 h with continuous stirring. Next, the suspension was filtered through sterile gauze; the flow-through was collected and washed three times with sterile saline, in between centrifugations for 10 min at 1,800× *g* at 4 °C. This resulted in a 3–10 ml pellet. To prevent carry-over contamination during processing, knives, all utensils and the food processor were thoroughly washed between samples with a detergent solution, followed by decontamination with a 10% hypochlorite solution and finally rinsing with distilled water.

### Mice (for bioassay)

For parasite isolation, female Swiss-Webster mice (Medical Military Academy Animal Research Facility, Belgrade) were used. Mice, weighing 18–20 g at the beginning of each experiment, were housed at the Institute for Medical Research Animal Research Facility at two per cage, and offered regular mouse feed and drinking water *ad libitum*. Naïve mice are occasionally examined for *T. gondii* infection at random for various purposes, and none were ever shown to be infected.

### Bioassay

Bioassays were performed as described previously [25]. Briefly, 1 ml of the heart tissue digest pellet supplemented with gentamicin was inoculated intraperitoneally (i.p.) into two naïve mice each per heart. Mice were monitored daily over a period of 6 weeks; peritoneal fluid of those that needed to be euthanized was examined for the presence of *T. gondii* tachyzoites, and microbiological culture of peritoneal fluid was performed. After 6 weeks mice were euthanized, blood samples taken for serology, and brains homogenized with 1 ml of saline each for cyst enumeration and, in case of positive findings, for DNA extraction, and subinoculation for further propagation. Cysts were counted under a phase-contrast microscope in four 25 μl drops of the brain suspensions, giving a threshold sensitivity of our method of 10 cysts/ml of brain homogenates. A bioassay was considered positive if at least one *T. gondii* cyst was detected in either mouse.

The remaining homogenate from cyst negative bioassays was frozen, while homogenates from cyst positive bioassays were prepared for *per os* inoculation into 4 naïve mice (2 to 5 cysts per mouse) for subsequent passage and strain genotyping.

The bioassay protocol used was approved by the State Ethics Committee (Veterinary Directorate of the Ministry of Agriculture and Environmental Protection of Serbia decision no. 323-07-02446/2014-05/1) and a local Ethics Committee (0313-1/11).

### DNA extraction and microsatellite analysis and Neighbour-joining clustering

Strains which were successfully established after initial bioassay and 1st passage were genotyped using MS analysis. Briefly, total genomic DNA was extracted from 100 μl of mouse brain homogenates using the NucleoSpin® Tissue (mini) kit (Macherey-Nagel, Düren, Germany), according to the manufacturer’s instructions. The resulting gDNA was re-suspended in a total volume of 100 μl of elution buffer and stored at -20 °C until shipment to BRC in Limoges. The isolates were genotyped based on length polymorphisms of 15 MS markers distributed on 10 of 14 chromosomes, as described previously [26].

For comparison, we included the genotyping data of all 61 strains with type III or type III-like genotypes (only excluding clones, i.e. repeat genotypes within the same locality), available at the BRC from Limoges, collected in Europe, the Americas, Africa/Middle East and Asia/Oceania (see Additional file 1: Table S1), seven of which have not been published previously. These 61 strains were collected from different animal species, or were isolated from patients with congenital toxoplasmosis (CT) by the French NRC Toxo. An unrooted neighbour-joining tree was reconstructed from MS data with Populations 1.2.32 (http://bioinformatics.org/populations/) based on Cavalli-Sforza and Edwards chord-distance estimator [27] and generated with MEGA 6.05 (http://www.megasoftware.net/history.php).

### Statistical analysis

For the analysis of serological results, 95% confidence intervals were calculated. The influence of examined variables on *T. gondii* seropositivity was analysed by Chi-square test. The possible influence of age (expressed in 5 two-year increments) on *T. gondii* seropositivity was analysed by Spearman’s rank correlation.

The level of significance was 5%. All statistics were performed using the SPSS version 11.5 statistical package (SPSS Inc., Chicago, IL, USA).

## Results

**Fig. 1:**
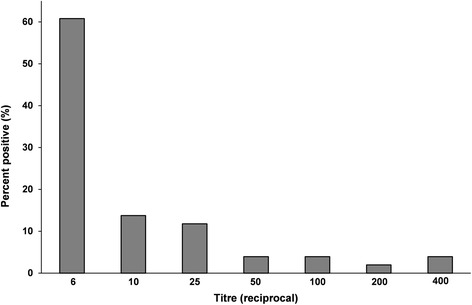
Distribution of *Toxoplasma gondii* antibody levels in seropositive slaughter horses in Serbia

The study series involved a total of 105 slaughter horses of both sexes ranging from 10 months to 9 years of age (mean 6.1 ± 1.8 years, median 6 years). The sampled population size represents over 13% of the estimated total number of horses slaughtered in Serbia during the sampling period. Seropositivity to *T. gondii* infection taking the lowest serum dilution (1:6) as the cut-off value was 48.6% (51/105), with the highest titre being 1:400 (Fig. 1). However, by raising the cut-off value to the 1:25 dilution, the seropositivity decreased to 12.4% (13/105).

Analysis of the zoographic variables showed that neither age group, gender, type/use, region nor slaughterhouse (place of sampling) had any influence on *T. gondii* seropositivity, as estimated at both the 1:6 (Table 1) and 1:25 cut-off (data not shown). The observed inter-regional differences (with the highest seropositivity recorded in Western Serbia) were not significant, presumably due to the small subgroups. There was no influence of age (rank) of the horses, either (Spearman rank correlation: *ρ*_(103)_ = -0.0398, *P* = 0.687).

**Table 1:** *Toxoplasma gondii*-specific antibodies as determined by the modified agglutination test (cut-off 1:6) in slaughter horses in Serbia according to zoographic characteristics

Variable	*n*	Seroprevalence (%)	95% CI	Chi-square *P*-value
Age group				
Yearling (1–2 years)	4	25.0	0–67.4	0.309
Young (3–4 years)	14	64.3	39.2–89.4	
Adult (5–9 years)	87	47.1	36.6–57.6	
Gender				
Female	51	43.1	29.5–56.7	0.279
Male	54	53.7	40.4–67	
Type/use				
Grade	79	50.6	39.6–61.6	0.723
Working	8	37.5	3.9–71	
Working, heavy	18	44.4	21.4–63.3	
Region				
Northern Serbia	10	30.0	1.6–58.4	0.233
Western Serbia	10	70.0	41.6–98.4	
Central Serbia	13	30.8	5.7–55.9	
Eastern Serbia	16	43.7	19.4–68.1	
Undetermined/Traders’	56	53.6	40.5–66.6	
Slaughterhouse				
A	56	51.8	38.7–64.9	0.481
B	49	44.9	31–58.8	
Total	105	48.6	39–58.1	

Bioassays were completed for 104 of the 105 horses, because in one bioassay both mice died on day 2 and 3 post infection, respectively, with large numbers of *Streptococcus equi* and *Pseudomonas* spp. bacteria cultured from the peritoneal exudates (pex). *T. gondii* tissue cysts were detected from two grade type mares of “undetermined” origin, slaughtered at the same abattoir but on different days. Interestingly, one of these tested negative for *T. gondii* antibodies, and the other was positive only at the 1:6 dilution (Table 2). All three mice harbouring detectable *T. gondii* brain cysts were seropositive at high titres (≥ 1:5120), while, conversely, no specific antibodies were detected in any of the mice from cyst-negative bioassays. Both strains (RS-Eq39 and RS-Eq40) were successfully passaged after initial isolation and have since been maintained at the NRL Toxo. Of note, both displayed an increased lethality for mice with successive passages. Although all mice inoculated with horse heart digests survived without clinical symptoms for 6 weeks while developing hundreds of cysts, mice started succumbing to the infection as of the first subsequent passage. The inoculum was thereafter lowered to 3 then 2 cysts for RS-Eq39, and to 4 and 3 cysts for RS-Eq40.

**Table 2:** Source animal characteristics and strain mouse bioassay and genotyping results of the two *Toxoplasma gondii* strains isolated from the hearts of slaughter horses in Serbia

Gender	Age	Type/use	MAT (horse sera)	Bioassay				Genotype
				Mouse 1		Mouse 2		
				Brain cysts/ml	MAT	Brain cysts/ml	MAT	
Mare	6	Grade	< 1:6	1010	1:20480	320	1:20480	III
Mare	4	Grade	1:6	690	1:5120	0	< 1:20	III

### Microsatellite analyses

**Fig. 2:**
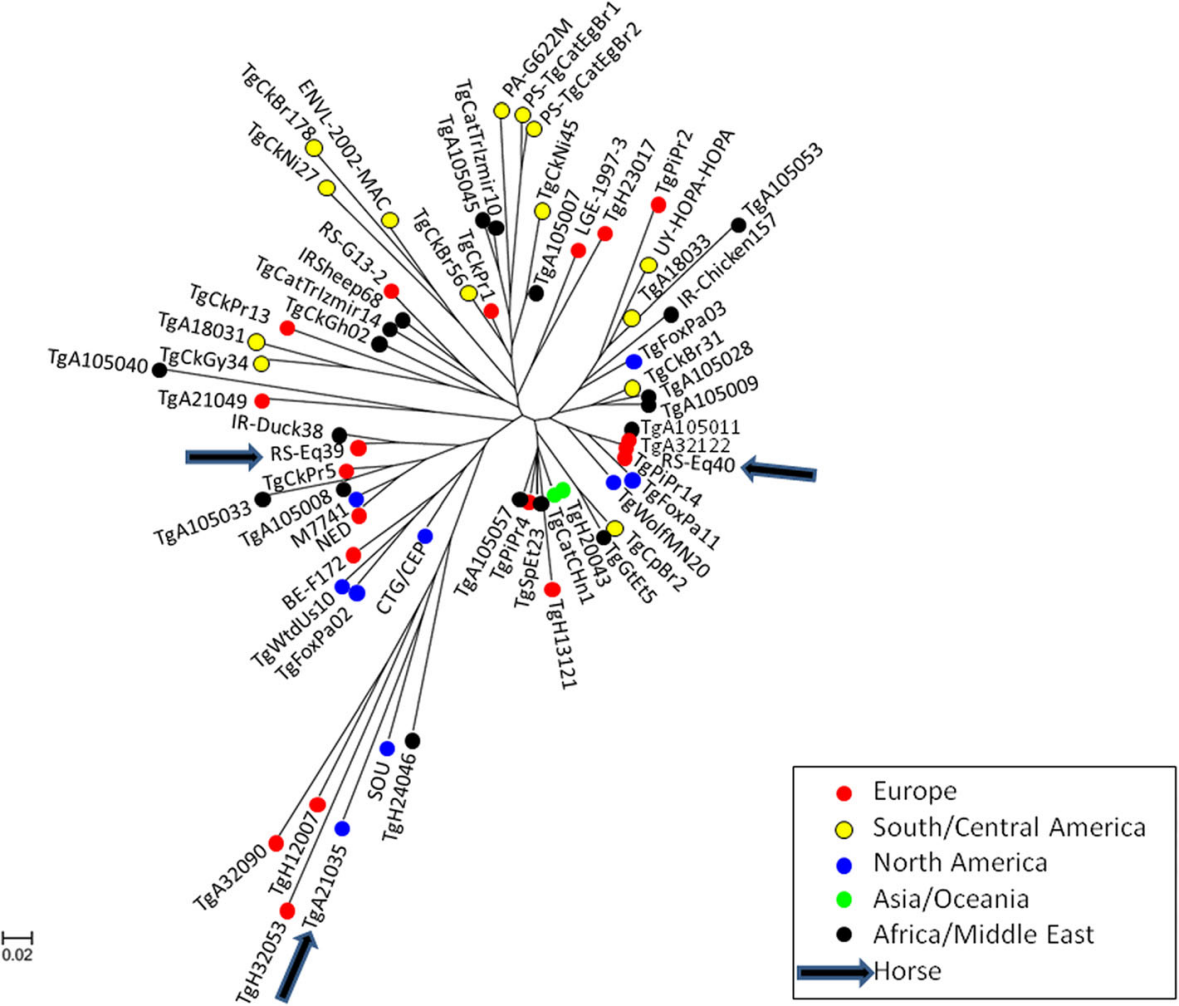
Neighbour-joining clustering of *Toxoplasma gondii* isolates based on 15 microsatellite markers. *Red* points are for isolates from Europe (*n* = 19); *yellow* points are for isolates from South and Central America (*n* = 14); *blue* points are for isolates from North America (*n* = 9); *green* points are for isolates from Asia and Oceania (*n* = 2); *black* points are for isolates from Africa and the Middle East (*n* = 19); arrows are for strains isolated from horses: two in this study (RS-Eq39 and RS-Eq40) and one in a previous study (TgA21035)

MS genotyping analysis showed both isolates to be type III. These strains were compared to a collection of 61 type III and type III-like strains available at the BRC from Limoges (Additional file 1: Table S1). The RS-Eq39 strain showed less frequent alleles for markers M48 and N61 than most type III strains isolated in Europe, and was most closely related to a strain isolated from a duck in Iran, IR-Duck38. The strain RS-Eq40 was identical to strains TgA105011 (goat from Gabon), TgA32122 (sheep from France) and TgPiPr14 (pig from Portugal) in all MS marker lengths (Additional file 1: Table S1). Figure 2 depicts an unrooted tree obtained by neighbour-joining analysis of the 63 strains.

Interestingly, both RS-Eq39 and RS-Eq40 differ from the other mentioned type III strain previously isolated from a pigeon in Serbia, RS-G13, which showed uncommon alleles for markers N60 and M102 with respect to the other strains isolated in Europe and was actually most closely related to a strain isolated from a sheep in Iran (IR-Sheep68) (Fig. 2).

Comparative MS marker analysis of genotypes of all 63 type III and type III-like isolates from Europe, the Americas, Africa, Asia and Oceania (Additional file 1: Table S1) revealed 59 distinct genotypes. Of the 63 analysed strains, a total of three were isolated from horses, which, in addition to the two from Serbia, include an isolate from horsemeat (TgA21035) imported from Canada into France [28].

## Discussion

This study showed a *T. gondii* seropositivity in horses from Serbia of almost 50% at a 1:6 MAT cut-off value, and 12.4% at 1:25 as the cut-off value. The value obtained at the 1:25 cut-off is significantly lower than the 30.8% seroprevalence found in an earlier study [14] (performed with the same test at the same cut-off) and probably reflects the difference in the age of the horses, since the animals in the present study were significantly younger (*P* = 0.00056). Worldwide, the reported seroprevalence in horses ranges from 0 to 100%, and from 0 to 53% in Europe (rev. in [29, 30]). Recent studies in Europe have shown seropositivity rates of 1.7% in Greece [31], 10.8% in Spain [32], 3% [33] and 17.6% in Italy [34], 23% in the Czech Republic [35], 37.8% in Romania [36], and from 13 to 90% (depending on the cut-off) in France [37]. It is difficult to assess whether the reported data represent real differences in the seroprevalence of *T. gondii* infection or possibly reflect the use of different assays, different cut-offs for modified agglutination assays or perhaps intrinsic differences in the study samples regarding age and/or true origin of the animals.

Despite numerous serological data, reports of *T. gondii* isolations from naturally infected horses are quite rare. Interestingly, first isolations come from 50 years ago [38–40], but after a long gap, the last few years have seen a renewed interest in this issue. In Brazil, *T. gondii* was detected in the brains of 3.5% (14/398) horses by mouse bioassay, according to presence of parasites in two mice (in brain and pex one each) or mouse IFAT seropositivity alone (in 12 cases) [41]. In Europe, two strains have only recently been isolated from the hearts of seropositive slaughter horses in Romania (by mouse bioassay), but were not genotyped [36]. In Italy, Papini et al. [34] have isolated three strains and genotyping by PCR-RFLP showed them to be of type I, II/III and III respectively. The isolation of two viable *T. gondii* strains from slaughter horses in Serbia described here is the first report in this country. It is interesting that both isolates belong to type III.

Archetypal type III and type III-like strains or DNA have been obtained from animals and human CT cases in Europe, and throughout the world (Additional file 1: Table S1). The two strains from Serbian horses are of archetypal type III, with the RS-Eq40 isolate sharing an identical MS genotype with strains with a very different geographical origin, within or from out of Europe (Gabon: TgA105011, France: TgA32122, Portugal: TgPiPr14), while the RS-Eq39 was most closely related to a strain isolated from a duck in Iran, IR-Duck38 (Additional file 1: Table S1; Fig. 2). This observed similarity between geographically distant strains may reflect recent clonal expansions of the Type III. On the other hand, in comparison to the only other horse isolate TgA21035 (a type III-like, from Canada), our two strains show differences on even 6 MS markers (B18, M48, M102, N60, AA, N83), demonstrating a different origin of these strains.

A worldwide distribution of type III *T. gondii* strains may be attributed to the proposed ancestral nature of type III relative to the other archetypal lineages [42, 43]. In Europe type III has mostly been isolated in Mediterranean countries (Additional file 1: Table S1) [44–49]. Its more frequent representation in the Mediterranean perimeter is further suggested by the present isolation of type III strains from horses and a previous one from a feral pigeon in Serbia [20]. These findings point to the possibility of importation of strains, sometime in the distant past, perhaps originally from Africa, where type III is common both in urban and rural environments [3, 43]. Moreover, type III strains were isolated, more frequently than type II strains, from birds in Egypt and Iran [50, 51]. It is therefore possible that migratory birds have contributed to the spread of type III *T. gondii* into Europe. A more recent factor that may facilitate the spread of *T. gondii* of different genotypes throughout the world is globalization of trade and transportation.

The results of this study also present interesting information on the virulence of the isolated type III strains. The isolates themselves were initially of a weakly virulent phenotype; such type III strains have been described in Gabon (TgA105011 equals GAB1-2007-CAP-AEG6 in Mercier et al. [43]) and French Guiana (A. Mercier, unpublished data). However, our horse isolates seem unique in that their virulence increased in subsequent oral passages. An increase in *T. gondii* pathogenicity in mice has been historically documented following frequent i.p. passage, notably with type III strains [52], but oral inoculation has frequently led to a decrease in virulence [25].

Data regarding the risk of transmission of *T. gondii* from infected horse meat to humans are scarce and inferred. In France, Pomares et al. [28] described three cases of human toxoplasmosis, in which MS genotyping showed all three to be different atypical strains, and epidemiologic explorations revealed that the probable source of infection was eating raw horsemeat, in two cases imported from Canada and Brazil, respectively. The risk was further emphasized by the isolation of a type III-like strain from horsemeat (originating from Canada) obtained from the first patient’s butcher [28]. Using MC-PCR, Aroussi et al. [37] detected *T. gondii* DNA in 43% of 231 horsemeat samples from French supermarkets. Strain isolation was attempted from 118 samples by mouse bioassay, but no parasites were isolated, which the authors considered to reflect low distribution of cysts in skeletal muscles, therefore indicating a low risk of human *T. gondii* infection from consuming infected horsemeat [37]. However, quite the opposite was concluded in a study of the relative contribution of sheep, beef and pork products to human *T. gondii* infection in the Netherlands (as quantified by Quantitative Microbial Risk Assessment), which showed a high public health risk even in the case of low prevalence of parasite cysts in animal tissues, if the meat is inadequately processed [53]. Therefore, as horsemeat is generally consumed rare or undercooked, the risk for human infection may be high even in the case of low cyst burden in horse tissues.

In the case of isolations in Serbia, a low cyst burden may indeed explain the low isolation rate in view of the high seroprevalence, at least as estimated according to the low MAT cut-off [54]. However, a low cut-off of 1:10 was shown to give the best sensitivity/specificity balance between the MAT test and the presence of *T. gondii* DNA in meat samples as detected by MC-PCR [37]. Another important point is that the strains we isolated originated from one weakly seropositive animal and from another completely seronegative one. This, however, agrees with the results of a recent systematic review which has shown very low agreement between serological results and the presence of *T. gondii* cysts especially in large animals including cattle and horses, with a low recovery rate in seropositives and similar rates of direct detection of the parasite in seronegative and seropositive animals [23].

## Conclusions

The isolation of viable *T. gondii* parasites from slaughter horses points to horsemeat as a possible and even probable source of human infection, a conclusion recently reached by a systematic review [55] as well. Moreover, successful isolations from horses with feeble serological evidence of *T. gondii* infection seem to confirm the notion [37] that the use of serological tests may not be recommended to assess the risk of human toxoplasmosis from consumption of horsemeat, which further limits the currently available tools to detect animals that may pose a threat to human health. Given that horsemeat tends to be consumed rare, the role of slaughter horses as sources of *T. gondii* infection should not be disregarded. Presence of *T. gondii* type III in Serbia sheds more light into the potential origin of this archetypal lineage in Europe.

## Additional file

Additional file 1: Table S1. Genotyping data of 63 *Toxoplasma gondii* strains with type III or type III-like genotypes collected in Europe, the Americas, Africa, Asia and Oceania. (XLSX 19 kb)

## Abbreviations

95% CI: 95% confidence interval
BRC: Biological Resource Centre *Toxoplasma*
CT: Congenital toxoplasmosis
DNA: Deoxyribonucleic acid
i.p.: Intraperitoneal
IFAT: Immunofluorescence antibody test
MAT: Modified agglutination test
MC-PCR: Magnetic capture-polymerase chain reaction
MS: Microsatellite
NRC Toxo: National Reference Centre for Toxoplasmosis (France)
NRL Toxo: National Reference Laboratory for Toxoplasmosis (Serbia)
PCR-RFLP: Polymerase chain reaction-restriction fragment length polymorphism
